# Obituary

**Published:** 1851-09

**Authors:** 


### OBITUARY.

Died, at Elgin, Ill., Aug. 6th, of Consumption, Dr. Erastus Sanford, in the 31st year of his age.

The highest testimonials of his geod stahding in the profession and in society, have been furnished.

